# Luteoloside Exerts Analgesic Effect in a Complete Freund’s Adjuvant-Induced Inflammatory Model *via* Inhibiting Interleukin-1β Expression and Macrophage/Microglia Activation

**DOI:** 10.3389/fphar.2020.01158

**Published:** 2020-07-31

**Authors:** Chun-Yan Shi, Xi-Biao He, Chao Zhao, Hui-Jing Wang

**Affiliations:** ^1^ Institute of Chinese Medicine, Shanghai University of Chinese Medicine, Shanghai, China; ^2^ Laboratory of Neuropsychopharmacology, College of Fundamental Medicine, Shanghai University of Medicine & Health Science, Shanghai, China; ^3^ National Clinical Research Center for Aging and Medicine, Huashan Hospital and MOE/NHC/CAMS Key Lab of Medical Molecular Virology, School of Basic Medical Sciences, Fudan University, Shanghai, China; ^4^ Graduate School, Shanghai University of Traditional Chinese Medicine, Shanghai, China

**Keywords:** analgesic effect, inflammatory pain, luteoloside, interleukin-1β, macrophage/microglia

## Abstract

**Background:**

Flavonoid monomers are proved to have an anti-inflammatory effect and may also be promising for chronic pain treatment. In the present study, the analgesic effect and the relevant mechanisms of luteoloside, one of the flavonoid monomers, were investigated.

**Methods:**

The analgesic effect of luteoloside was first evaluated in complete Freud’s adjuvant induced inflammatory model by von Frey test and Hargreaves test in both male and female mice. The interleukin-1β levels in plantar tissue, serum, dorsal root ganglion, and the dorsal horn of the spinal cord were determined by enzyme-linked immunosorbent assay or immunofluorescence. The activation of macrophage/microglia was tested by Iba-1 staining.

**Results:**

Our data showed that luteoloside exhibited both acute and chronic analgesic phenotypes. Every single dose of luteoloside solution reached the peak transient analgesic effect 2 h after administration and lasted less than 6 h. About 14 consecutive days administration (one dose per day) later, luteoloside showed a sustained analgesic effect which lasted more than 24 h. Celecoxib 20 mg/kg combined with luteoloside 40 mg/kg achieved a similar analgesic effect as celecoxib 40 mg/kg alone. Luteoloside inhibited interleukin-1β expression in plantar tissue, dorsal root ganglion, the dorsal horn of spinal cord, and serum, after 14 days of continuous administration. Furthermore, our results also showed that the activation of macrophage/microglia in dorsal root ganglions were significantly inhibited 2 h after each single dose in daily luteoloside administration and recovered to a higher level 6 h later. These findings might be involved in the mechanisms of the acute analgesic effect of luteoloside.

**Conclusion:**

Luteoloside presents an analgesic effect *via* anti-inflammatory and other mechanisms such as inhibiting the activation of macrophage/microglia.

## Introduction

The presence of inflammation is one of the major underlying causes of chronic pain (Lipnik-Stangelj, 2013). In general, inflammatory stimulus leads to the production of pro-inflammatory mediators, such as interleukin-1β (IL-1β), IL-6, tumor necrosis factor α (TNF-α), and Prostaglandin E_2_ (PGE_2_) (Owens et al., 2005; Tosun et al., 2014). That may occur due to the activation of both nociceptors and macrophage/microglia. Macrophage/microglia cells are known to be involved in inflammation since they are the critical immune cells in the nerve system (Rawji et al., 2016; Wolf et al., 2017). They secrete a variety of pro-inflammatory cytokines, as well as inducing the expression of cyclooxygenase-2 (COX-2) which leads to the release of prostanoids (Samad et al., 2001; Hanisch, 2002; Lees et al., 2015; Vicario et al., 2016; Haight et al., 2019). These inflammatory factors may regulate hyperalgesia or allodynia *via* multiple signaling pathways. For example, the inflammatory response mediated by LPS is mainly activated by nuclear factor-kappa B (NF-κB) and mitogen-activated protein kinase (MAPKs) pathway (Chun et al., 2012). The levels of phosphorylated extracellular signal-regulated kinase1/2 (ERK1/2) and p38 MAPK in DRG tissues were increased one day after the Complete Freund’s Adjuvant (CFA) modeling (Svensson et al., 2003; Ji et al., 2018). Intraplantar microinjection of carrageenan and CFA could potentiate the Nav1.3, Nav1.7, and Nav1.8 channels to produce inflammatory pain (Baker and Bostock, 1997). PGE_2_ can reliably increase the Nav1.9 channel current in mice DRG neurons with G-protein activation (Rush and Waxman, 2004). These studies suggest that suppression of inflammation is essential to pain-relieving.

In recent years, many flavonoids were found to have anti-inflammatory effects (Spagnuolo et al., 2017). Among these flavonoids, a monomer called luteoloside has attracted the attention. Luteoloside (also called cynaroside or luteolin-7-O-glucoside) is commonly found in *Lonicera japonica Thunb*, as well as other plants, such as lettuce, *Glossogyne tenuifolia, Salvia plebeian* (Wu et al., 2004; Ha et al., 2006; Akram et al., 2015; Cui et al., 2017; Lin et al., 2019). According to recent studies, luteoloside has various physiological and biochemical effects, such as anti-oxidative, anti-inflammatory, antibacterial, antiviral, anticancer, and other functions (Qiu et al., 2013; Xiong et al., 2013; Song and Park, 2014; Wang et al., 2018a). It has been reported that luteoloside exhibited an anti-inflammatory effect on lipopolysaccharide (LPS)-induced inflammatory model. In this study, luteoloside reduced the secretion of inflammatory cytokines, including interleukin-1β (IL-1β), which derived by LPS-mediated macrophages (Song et al., 2017). Some reports showed that luteoloside alleviated neurological impairment and cerebral edema, as well as improved cerebral infarction and histopathological changes in middle cerebral artery occlusion (MCAO) rats. Luteoloside significantly inhibited cerebral ischemia-reperfusion (I/R)-induced neuroinflammation, as demonstrated by reduced levels of IL-1β in the brain tissues of MCAO rats (Li et al., 2019). When the uterus is infiltrated by *Staphylococcus aureus*, luteoloside could decrease the expression of the pro-inflammatory cytokines IL-1β *in vivo* and *in vitro* (Wang et al., 2018a). Besides, recent studies found *Glossogyne tenuifolia* extract attenuates inflammatory mediator synthesis partly through down-regulating NF-κB activation (Wu et al., 2004; Ha et al., 2006). What is more, luteoloside can inhibit cell proliferation *via* mitogen-activated protein kinase (MAPK) and mammalian target of rapamycin (mTOR) signaling pathways (Shao et al., 2018). The anti-inflammatory effect of luteoloside and its effects on pain-related signaling pathways indicate its application prospect in chronic inflammatory pain. However, there is no data about the analgesic effect of this drug until now. In the present study, we tested the hypothesis that luteoloside relieved pain *via* an anti-inflammatory mechanism.

Analgesics are in great clinical need. The most popularly used ones are non-steroidal anti-inflammatory drugs (NSAIDs). However, with the increase in the dosage of these drugs, severe adverse reactions of the digestive system could occur (Gargallo and Lanas, 2013; Scarpignato et al., 2015), which limits the application of these drugs in chronic pain. New selective COX-2 inhibitors (e.g., celecoxib) were developed as an alternative to reduce these risks (Zeng et al., 2018). However, severe adverse reactions of the cardiovascular system still limit their clinic application. Ye Won Jeon et al. found the combination of celecoxib and luteolin induced synergistic effects *via* ERK signaling inhibition in cancer cells (Jeon et al., 2015). Although these data were not achieved in studies about analgesic effects, due to the critical roles of inflammation and MAPK signaling pathway in pain (Svensson et al., 2003; Ji et al., 2009), we further investigated the synergistic effect of luteoloside and celecoxib on CFA-mediated inflammatory pain. The results of the present study indicate a potential application of luteoloside as an analgesic in the clinic.

## Materials and Methods

### Animals

Male and female C57BL/6 mice aged 12–14 weeks were used. Mice were maintained in the animal facility of the Shanghai University of Medicine & Health Science (SUMHS). All experiments were performed in accordance with the international guidelines and approved by the animal ethics committee of SUMHS (No. 2018-GJZDYFJH-08-054629). All experiments were performed in a blinded set-up.

### Drugs

To producing an inflammatory pain model, complete Freund’s adjuvant (CFA, Sigma-Aldrich, 10 μL) was injected intraplantarly. Luteoloside (HPLC≥98%, Shanghai Yuan Ye Biotechnology Co., Ltd) were dissolved in 1% DMSO (Sigma-Aldrich), and the concentration was adjusted to dosages of 20, 40, and 80 mg/kg by intraperitoneal injection, respectively. Celecoxib (HPLC≥98%, Shanghai Yuan Ye Biotechnology Co., Ltd) was dissolved in saline and adjusted to dosages of 20 and 40 mg/kg by intraperitoneal injection. All the drugs were prepared just before use.

### Mechanical and Thermal Hyperalgesia

Mechanical allodynia was measured by using von Frey hairs as described before (Willemen et al., 2010; Wang et al., 2018b). In brief, before experiments, mice were exposed to the equipment without any nociceptive stimulation for 1–2 h/d for 3 d. On day 3, mice were placed in the test environment 15–20 min before testing. Baseline responses were determined on three different days, and the 50% paw withdrawal thresholds were calculated using the up-and-down method (Chaplan et al., 1994).

Thermal hyperalgesia was determined by measuring Paw Withdrawal Latencies (PWLs) using Hargreaves radiate heat apparatus (IITC Life Science) as described before. The intensity of the light beam was chosen to induce a heat withdrawal latency time of approximately 10 s at baseline. A cut-off time of 15 s was set to prevent tissue damage (Hargreaves et al., 1988).

### Immunofluorescent Staining of DRGs and Spinal Cord

Mice were deeply anesthetized with an overdose of sodium pentobarbital and transcardially perfused with PBS followed by 4% paraformaldehyde, after which lumbar DRG (L3–L5) and spinal cord (enlargement) were collected. Tissues were post-fixed in 4% paraformaldehyde for 4 h and then cryoprotected in 20% sucrose in PBS at 4 °C, followed by 30% sucrose in PBS overnight. The tissues were then embedded in OCT compound and frozen at −80 °C. Frozen sections of DRG and spinal cord (14 μm) were washed with 0.01 M PBS and then 0.03% PBS TritonX-100, they were incubated with the 5% bovine serum albumin (BSA, VWR Life Science) for 2 h at room temperature. Sections were then incubated overnight at 4 °C with rabbit anti-Iba-1 (1:200, Abcam), or rabbit anti-IL-1β (1:200, Abcam), respectively. After washed with 0.03% PBST, the sections were then incubated with Alexa Fluor-488 donkey-anti-rabbit antibody (1:500, Invitrogen).

The sections were photographed with DFC450 C (Leica Microsystems Ltd.) using identical exposure times for all slides. Count of Iba-1 positive staining cells in DRG and spinal dorsal horn were analyzed with NIH ImageJ. The numbers of Iba-1 positive staining cells per square millimeter were then calculated. The IL-1β staining in the DRG and spinal cord horn were photographed with fixed exposure times, and IL-1β expression levels were analyzed with NIH ImageJ (Nahid et al., 2019). All stainings were done in parallel for different groups. The average background fluorescence for control staining sections were subtracted before calculation of the IL-1β staining.

### Enzyme-Linked Immunosorbent Assay (ELISA)

Plantar tissue and serum were obtained at different time points and were immersed in liquid nitrogen immediately. The samples were stored at −80°C until analysis.

The plantar tissue and serum samples were subjected to measurement of IL-1β level using ELISA kits (IL-1β, R&D Systems) according to the manufacturer’s instructions. The results were calculated using interpolation from a standard curve created by a series of standard IL-1β concentrations.

### Statistics

Data are expressed as mean ± SEM. Statistical analysis was carried out using one-way or two-way ANOVA followed by Bonferroni analysis. A *P* value of less than 0.05 was considered to be statistically significant.

## Results

### Luteoloside Produced an Analgesic Effect in the CFA-Induced Inflammatory Pain Model

**Figure 1:**
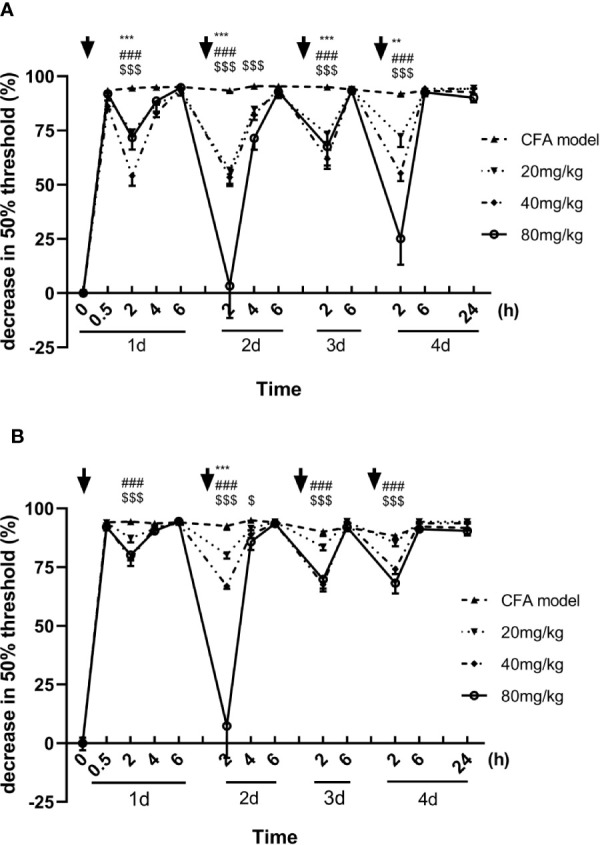
Effect of luteoloside on mechanical allodynia in male **(A)** and female **(B)** Complete Freund’s Adjuvant (CFA)-model mice. Mice received a dose of luteoloside every day for 4 consecutive days after CFA modeling. The changes in 50% paw withdrawal thresholds were measured with von Frey hair over time. Data are expressed as mean ± SEM. n = 8/group. **p < 0.01, ***p < 0.001, 20 mg/kg group *v.s.* CFA model group; ^###^ *P* < 0.001, 40 mg/kg group *v.s.* CFA model group; ^$^ *P <*0.05, ^$$$^ *P < *0.001, 80 mg/kg group *v.s.* CFA model group.

Inflammatory hyperalgesia was induced in mice with an intraplantar injection of CFA. The 50% pain thresholds of the mice were decreased continuously after the intraplantar injection of CFA. With each single dose administration of luteoloside, the decrease of 50% mechanical pain thresholds were significantly improved and reached its peak effect at 2 h after luteoloside administration in both male and female mice, while such analgesic effect of luteoloside attenuated 6 h later (**Figures 1A, B**).

**Figure 2:**
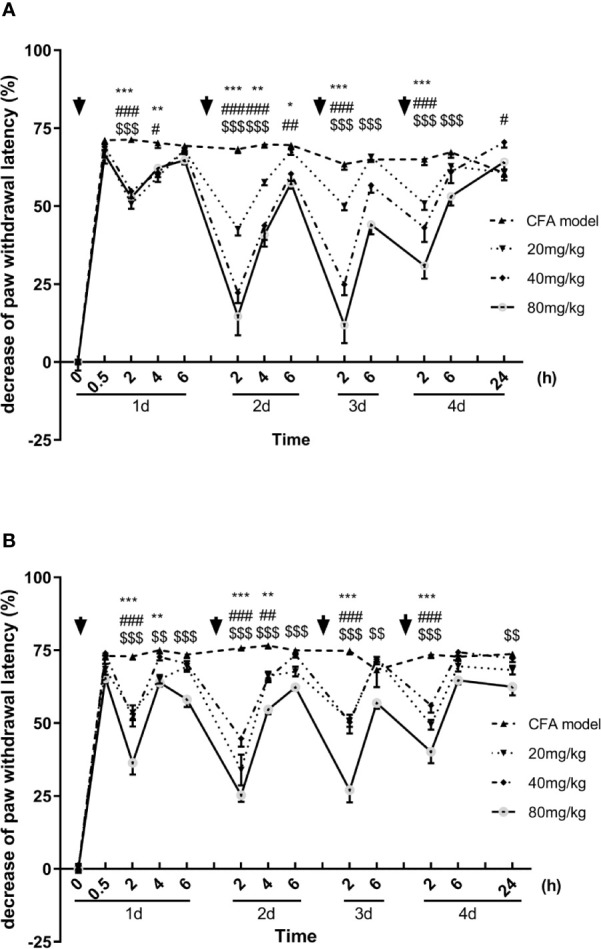
Effect of luteoloside on thermal hyperalgesia in male **(A)** and female **(B)** Complete Freund’s Adjuvant (CFA)-model mice. Mice received a dose of luteoloside every day for 4 consecutive days after CFA modeling. The paw withdrawal latency were measured with Hargreaves test over time. Data are expressed as mean ± SEM. n = 8/group. **p< 0.01, ***p< 0.001, 20 mg/kg group *v.s.* CFA model group; ^#^ *p*< 0.05, ^##^ *p*< 0.01, ^###^ *P* < 0.001, 40 mg/kg group *v.s.* CFA model group; ^$$^ *p*< 0.01, ^$$$^ *P <*001, 80 mg/kg group *v.s.* CFA model group.

Similar to the results of mechanical allodynia, the luteoloside prolonged the paw withdrawal latency in the Hargreaves test, showing an analgesic effect in the thermal hyperalgesia. With each single dose administration, the latencies were significantly prolonged rapidly and reached to peak effect at 2 h, the analgesic effect of luteoloside also attenuated 6 h later in both males and females (**Figures 2A, B**).

**Figure 3:**
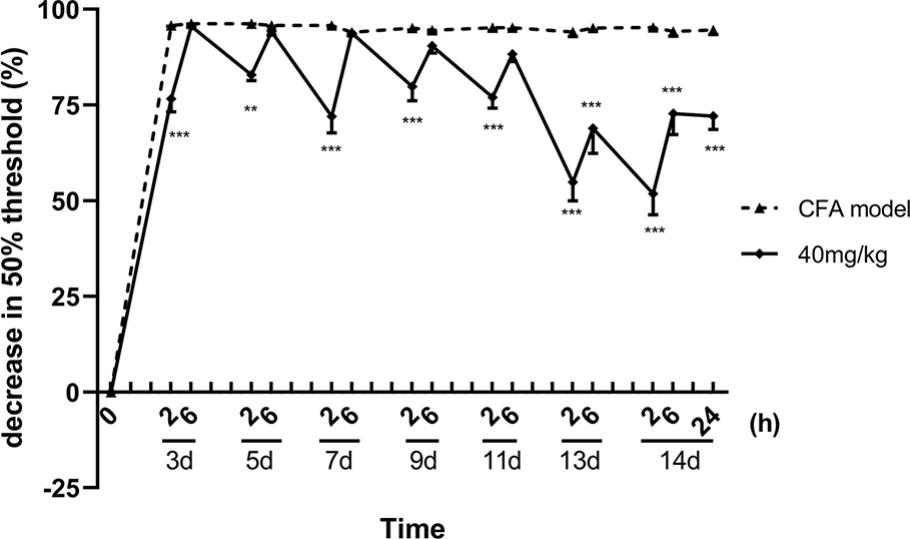
Sustained analgesic effect of luteoloside on mechanical allodynia in Complete Freund’s Adjuvant (CFA)-induced male mice. Mice received a dose of luteoloside every day after CFA modeling for 14 d in total. The changes in 50% paw withdrawal thresholds were measured with von Frey hair over time. Data represent mean ± SEM. n = 8/group. **p < 0.01, ***p < 0.001 *v.s.* CFA model group.

About 13 consecutive days administration (one dose per day) later, luteoloside showed a sustained analgesic effect, in addition to its rapid and transient effect, on mechanical allodynia in CFA pain model mice. Although the analgesic effect was weakened after 6 h as before, the 50% pain thresholds were still higher than the model control group 24 h later, suggesting the analgesic effect of luteoloside was maintaining (**Figure 3**).

These results indicated that a single dose of luteoloside produced a rapid and transient analgesic effect within 2 h after administration in these CFA pain model mice. With the repeated administration, luteoloside showed a more significant analgesic effect, and its duration is longer. The luteoloside had no effect on both of the mechanical pain thresholds and thermal paw withdraw of naive mice (data are not shown).

### Luteoloside Enhanced the Analgesic Effect of Celecoxib

**Figure 4:**
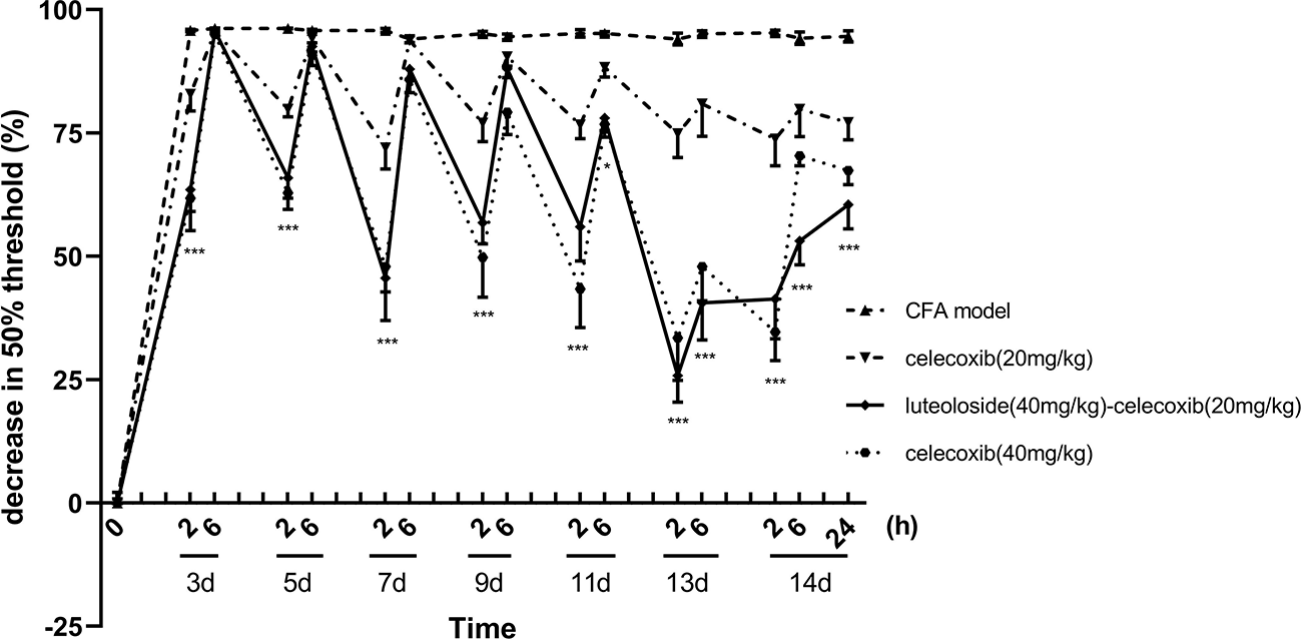
The synergistic analgesic effect of luteoloside combined with celecoxib. Mice received different treatments, respectively, every day after Complete Freund’s Adjuvant (CFA) modeling for 14 d in total. The changes in 50% paw withdrawal thresholds were measured with von Frey hair over time. Data represent mean ± SEM. n = 8/group. *p < 0.05, ***p < 0.001, luteoloside (40 mg/kg)-celecoxib (20 mg/kg) combined group *v.s.* celecoxib (20 mg/kg).

Celecoxib is a classical NSAIDs which is widely used for the relief of pain in the clinic (Mi et al., 2013; Li et al., 2016; Sriram et al., 2017). However, with the increase in dose, adverse reactions of the cardiovascular system and digestive system become obvious (Gargallo and Lanas, 2013; Scarpignato et al., 2015). In our present study, we compared the effect of luteoloside combined with celecoxib. The results showed that the analgesic effect of 20 mg/kg celecoxib combined with luteoloside produced a better analgesic effect than that of 20 mg/kg celecoxib alone. The analgesic effect of the combination group was comparable to that of celecoxib at 40 mg/kg (**Figure 4**).

### Luteoloside Inhibited the Release of IL-1β After CFA-Induced Inflammation

IL-1β is a critical inflammatory factor, which is related to pain (Dinarello, 2004; Menetski et al., 2007; Kawasaki et al., 2008; Ke and Richard, 2009). To test our hypothesis that luteoloside produces analgesic effect *via* anti-inflammation, we determined the levels of IL-1β at different time points and in different tissues.

**Figure 5:**
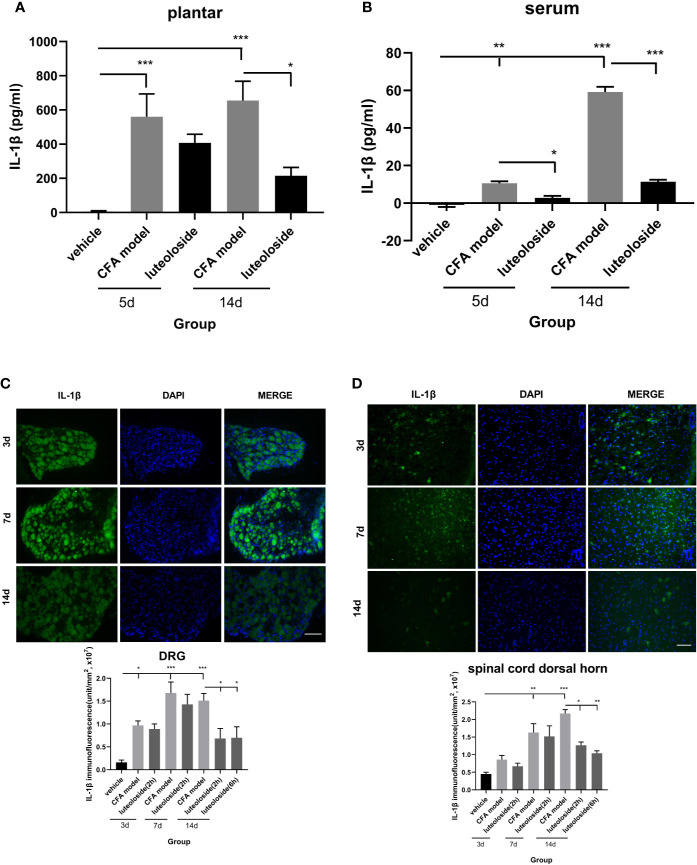
The changes of IL-1β levels in plantar **(A)**, serum **(B)**, dorsal root ganglions (DRGs) **(C)**, and spinal cord dorsal horn **(D)** at different time points. Mice received a dose of luteoloside every day after Complete Freund’s Adjuvant (CFA) modeling for 14 d in total. The levels of IL-1β at different time points in plantar, serum, DRGs and spinal cord dorsal horn were measured by ELISA or immunofluorescent staining. Data represent mean ± SEM. n=4/group. Scale bar=100μm. *p < 0.05, **p < 0.01, ***p < 0.001.

The release of IL-1β in plantar (**Figure 5A**) and dorsal root ganglion (DRG) (**Figure 5C**) increased rapidly after CFA injection and lasted for more than 14 d. While, the level of IL-1β in both serum (**Figure 5B**) and spinal cord dorsal horn (**Figure 5D**) increased gradually and reached a peak level after 14 d, indicating the inflammation progressively spread from local tissue and nociceptive nerve ending to the systemic and central nerve system.

Luteoloside inhibited the release of IL-1β after the CFA model, suggesting an anti-inflammation effect of luteoloside. The data of day 3~7 after luteoloside administration showed that the inhibition was weak during the initial stage of the drug treatment. There was no significant difference between the model group and luteoloside group in plantar, DRGs, and spinal cord dorsal horn (**Figures 5A, C, D**). With the prolongation of administration time, the inhibition of IL-1β releasing became more significant. On day 14 (once per day continuously), all the levels of IL-1β in plantar, serum, and notably in DRGs and spinal cord dorsal horn were decreased after luteoloside treatment (**Figures 5A–D**). This suggested an accumulation of the anti-inflammatory effect of luteoloside.

### Luteoloside Inhibited the Activation of Macrophage/Microglia in DRGs Rapidly and Transiently, Contributing to Its Analgesic Effect

**Figure 6:**
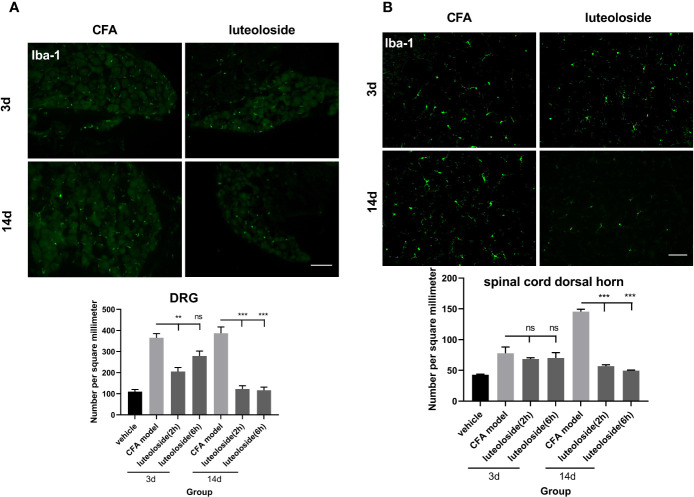
The activation level of macrophage/microglia in dorsal root ganglions (DRGs) **(A)** and spinal cord dorsal horn **(B)**. Mice received a dose of luteoloside every day after Complete Freund’s Adjuvant (CFA) modeling for 14 d in total. Iba-1 positive cells at different time points in DRGs and spinal cord dorsal horn in different groups are counted with ImageJ. Data represent mean ± SEM. n=4/group. Scale bar=100μm. **p<0.01, ***p<0.001.

The data of behavior experiments showed that luteoloside produced a rapid and transient analgesic effect 2 h after each administration and attenuated 6 h later. Such phenotype could not be explained by the cumulative anti-inflammatory effect of luteoloside on IL-1β production. We thus assumed that luteoloside exerted analgesic effect *via* multiple mechanisms besides anti-inflammation. The activation of macrophage/microglia was then examined. The results showed that the expression of Iba-1 in DRGs was rapidly increased and lasted for a long time after the CFA model. Strikingly, in the initial stage of luteoloside treatment (day 3), the expression of Iba-1 was significantly decreased 2 h after a single dose of luteoloside treatment, while it soon increased again 6 h later. After 14 d of continuous administration, luteoloside could continuously reduce the expression of Iba-1 in DRGs for 6 h (**Figure 6A**). While luteoloside had no effect on the expression of Iba-1 in spinal cord dorsal horn during the initial stage. It was not until after 14 d repeated administration that luteoloside continuously inhibited Iba-1 positive levels in the spinal dorsal horn (**Figure 6B**).

## Discussion

Luteoloside, a flavonoid that can be isolated from many plants, such as *Lonicera japonica*, exerts different biomedical and pharmacological activities, including anti-tumor (Nakashima et al., 2015), anti-bacterial (Xiong et al., 2013), anti-inflammation (Akram et al., 2015), and antiviral properties (Cao et al., 2016).

Here, the analgesic effect of luteoloside was first observed in the CFA-induced inflammation model. Luteoloside produced an analgesic effect in both CFA-induced mechanical allodynia and thermal hyperalgesia, and in both male and female mice. Luteoloside exhibited two different analgesic phenotypes. Single-dose of luteoloside showed a significant acute analgesic effect, which lasted only about 2 h. About 13 consecutive days administration (one dose per day) later, luteoloside showed a more sustained effect. These results suggested that luteoloside may exert an analgesic effect *via* different mechanisms. According to the present study, the different mechanisms probably are suppression of inflammation and inhibiting the macrophage/microglia activation.

As an active monomer component of flavonoids, luteoloside has been proven to have an anti-inflammatory effect in the previous studies (Song and Park, 2014). Our results of the changes in IL-1β levels also showed the anti-inflammatory effect of luteoloside *in vivo*. IL-1β is a pro-inflammatory cytokine that exerts a multi-effect on a variety of cells and plays a critical role in the acute and chronic inflammation (Kobayashi et al., 2005; Ke and Richard, 2009; Potter et al., 2010; Seul et al., 2018). It is known to be released by activated glia cells and neurons, contributing to persistent hyperalgesia and allodynia (Watkins et al., 2003; Thacker et al., 2007; Wang et al., 2017).

In the present experiments, the local IL-1β levels in the plantar and DRGs were rapidly increased after CFA injection, indicating that the local tissue and nociceptive nerve ending developed an acute inflammatory response with the CFA stimulation. At the first 3 to 5 d’ administration, luteoloside only decreased the serum IL-1β level lightly and did not affect the levels of IL-1β in the plantar, DRGs, and spinal cord dorsal horn. But luteoloside significantly inhibited IL-1β levels in these tissues after consecutive days of administration (one dose per day), showing an anti-inflammatory effect *in vivo*. Taken together, these results indicated that luteoloside had an anti-inflammatory effect, which may contribute to its long-term pain-relieving. This kind of anti-inflammatory effect can only be shown after the drug accumulated to a certain dose.

Strikingly, our results showed that a single dose of luteoloside showed a significant acute analgesic effect, which lasted only about 2 h. Four to six h later, the effect disappeared. These results suggested that in addition to inhibiting the expression of inflammatory factors, there is a transient mechanism of analgesic effect of luteoloside. According to V. Raghavendra et al., the mRNA level of IL-1β increased within 4 h after CFA modeling. However, the protein level of IL-1β did not increase significantly until 4 d after CFA modeling. Those results are similar to our data. Thus, the protein expressions of inflammatory factors are not quick enough to affect the change of pain thresholds within 4 h. Our study also showed that microglia are activated rapidly after modeling, which is simultaneous as the hyperalgesia had reached its peak, suggesting that microglia participate in the rapid regulation of hyperalgesia (Raghavendra et al., 2004; Tanga et al., 2004). Furthermore, a study by Berta et al. showed an acute action of microglia in regulating synaptic plasticity following noxious stimulation and acute inflammation (Berta et al., 2014). In our present study, the effect of luteoloside on the activation of microglia/macrophage in DRGs and spinal cord dorsal horn were evaluated. The activity of the microglia in the spinal cord dorsal horn was increased 3 d after modeling and reached a peak on day 14th. Such changes were consistent with the development of inflammation after CFA injection. Luteoloside had no effect on the microglia activities until 14 d continuously administration. On the 14th day after the luteoloside application, the activities of microglia in the dorsal horn of the spinal cord were continuously inhibited (at least 6 h). Combined with the effect of luteoloside on the level of IL-1β, we supposed that the inhibitory effect of luteoloside on microglia activities 14 d after treatment was related to its anti-inflammatory effect. The remarkable results appeared in the activities of macrophage/microglia in DRGs. The activities of macrophage/microglia in DRGs were significantly inhibited 2 h after luteoloside administration and recovered to a higher level 6 h later. These changes in time courses are similar to the changes in pain thresholds. We thus supposed that the transient inhibition effect of single-dose luteoloside on macrophage/microglia in DRGs might be involved in the mechanisms of its acute analgesic effect. The mechanisms of acute analgesia resulting from microglia inhibition are unclear yet. Studies suggest that some mechanisms, including adenosine-triphosphate (ATP) or some ion channels, may be involved in the rapid effect (Ji et al., 2018; Tsuda, 2018; Robinson et al., 2019; Tsuda, 2019).

Luteoloside could be a promising treatment of chronic inflammatory pain. In the present study, we showed not only the analgesic effect of luteoloside alone, but also the enhancement of the analgesic effect of celecoxib combined with luteoloside. The analgesic effect of celecoxib 20 mg/kg combined with luteoloside was comparable to that of celecoxib 40 mg/kg. Some studies have reported the side effects of celecoxib with dose-effect (Hubky et al., 2001; Levy and Fink, 2001; Gagnon et al., 2003). Four doses of celecoxib were used during the challenge: 20 g, 60 g, 120 mg, and 200 mg. Some patients developed a moderate angioedema of the lips about 40 min after administration of the single cumulative dose of 200 mg celecoxib, but the patients who received other three lower doses did not (Liccardi et al., 2005). In our present experiments, luteoloside combined with a lowered dose of celecoxib could achieve similar analgesic effect as a higher dose of celecoxib, suggesting the potential benefits of luteoloside. There are studies showed that IL-1β mediated the expression and activation of COX-2 and further promoted the release of PGE_2_ (Lin Shi et al., 2006; Samad et al., 2001). Our present data showed that luteoloside significantly reduced the level of IL-1β. That effect may further decrease the expression and activity of COX-2, and reduce the release of PGE_2_ in both DRGs and spinal cord. That may also be the reason for the synergistic effect of the combination of luteoloside and celecoxib, which is a COX-2 inhibitor. Moreover, luteoloside was found to have an acute analgesic effect in this study; meanwhile, celecoxib was also reported to have similar acute analgesic effect (Zhao et al., 2017). Some reports have found that luteoloside and celecoxib both have COX-2-independent effects on the signal pathways, such as MAPK/p38, and ERK. (Wu et al., 2004; Hua et al., 2005; Ha et al., 2006; Kadam et al., 2007; Park et al., 2010; Wang et al., 2013; Akram et al., 2015; Jeon et al., 2015; Li et al., 2019; Shao et al., 2018). Although these results have not been applied to studies about analgesic effects, these mechanisms may be the reason of the synergistic effect in the acute analgesic role due to the critical role of MAPK/p38 and ERK signal pathways in the pain regulation. Those results indicated that when combined with luteoloside in the clinic, the dosage of celecoxib can decrease to achieve a similar analgesic effect and reduce adverse effects.

Recent evidence showed sex dimorphism in pain processing by immune cells, primarily by microglia and macrophages (Sorge et al., 2015; Taves et al., 2016). For example, Sorge et al. demonstrated Spinal toll-like receptor 4 (TLR4) regulated inflammatory pain only in male but not female mice, highlighting possible male-dominant microglial signaling in the spinal cord dorsal horn (Sorge et al., 2011; Liu et al., 2016). Xin Luo et al. discovered that intrathecal injection of Resolvin D5 (RvD5) might target immune cells in DRGs for producing sex-dependent analgesic actions (Luo et al., 2019). Macrophage signaling in DRGs also appeared to regulate chemotherapy-induced peripheral neuropathy (CIPN) in a sex-dependent manner (Liu et al., 2014; Montague and Malcangio, 2017; Montague et al., 2018). Thus, whether luteoloside also produces the analgesic effect in female mice *via* similar mechanisms as in male mice needs more investigation.

## Data Availability Statement

All datasets generated for this study are included in the article/supplementary material.

## Ethics Statement

The animal study was reviewed and approved by The animal ethics committee of Shanghai University of Medicine & Health Science.

## Author Contributions

C-YS and H-JW designed the study, conducted the experiments, and drafted the manuscript. X-BH and CZ supervised the study, contributed to writing, and editing the manuscript. All authors contributed to the article and approved the submitted version.

## Funding

This work has been supported by the National Key Research and Development Program of China (2018YFC2002000) and the Scientific Research Foundation for Experts Recruitment by Shanghai University of Medicine & Health Sciences.

## Conflict of Interest

The authors declare that the research was conducted in the absence of any commercial or financial relationships that could be construed as a potential conflict of interest.
